# 524. Assessing the Safety of an Outpatient Remdesivir Infusion Program for Patients with Severe COVID-19 in the Setting of a Pandemic Surge

**DOI:** 10.1093/ofid/ofab466.723

**Published:** 2021-12-04

**Authors:** Benjamin Gee, Anita Cheruvanky, Graciela Faiad, Aldon Li, Huan Pham, Earl Quijada, Susan Sun, Adam Baghban

**Affiliations:** 1 Kaiser Permanente, Riverside, California; 2 Kaiser Permanente Riverside, Riverside, California

## Abstract

**Background:**

(i) Remdesivir (RDV) shortens recovery time among COVID-19 patients in an inpatient setting. (ii) Treatments for outpatients diagnosed with COVID-19 are limited. (iii) In early 2021, there was a national surge in COVID-19 hospitalizations, which resulted in hospital bed and staff shortages. (iv) In the face of this pandemic surge, we piloted a program to expand our RDV treatment capacity by establishing an off-label, outpatient infusion tent (OIT) for patients with severe COVID-19. (v) This is a retrospective, descriptive report examining the safety and efficacy of this program, with outcomes of interest being 30-day mortality and hospital admission within the subsequent 30 days

**Methods:**

(i) The OIT, consisting of 11 chairs capable of treating 35 patients per day, was operational from January 1 to February 19, 2021. (ii) Patients were referred to the outpatient RDV program primarily from urgent care (UC) and the emergency department (ED), and from the inpatient setting to complete therapy. Patients received at least one dose prior to referral. (iii) Eligibility criteria included a confirmed COVID-19 diagnosis, radiographic evidence of viral pneumonia, and an oxygen saturation less than or equal to 94 on room air. (iv) Exclusion criteria included pregnancy, sepsis, end-stage renal disease or GFR < 30, hepatitis with transaminases 10 times the limit of normal. Patients with BMI > 40, age > 75, chronic lung disease, dementia, were considered on a case by case basis. (v) Patients received dexamethasone and deep vein thrombosis prophylaxis

**Results:**

(i) A total of 88 patients received 258 infusions. The average number of outpatient infusions per participant was 2.9. (ii) Four out of 88 patients died (4.5%) within 30 days of first dose in the infusion tent. No deaths occurred in the outpatient setting. (iii) Fourteen out of 88 patients were admitted to the hospital within the subsequent 30 days (15.9%). (iv) 11/14 admissions (78.6%) were due to progression of COVID-19. There were no admissions due to adverse drug reactions

Table 1. Patient Characteristics

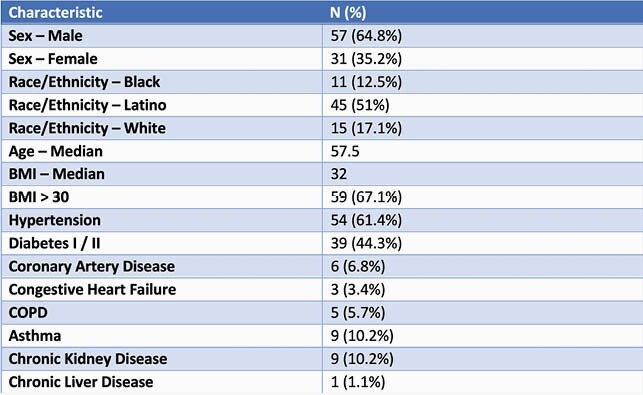

Table 2. Admissions Within Subsequent 30 Days

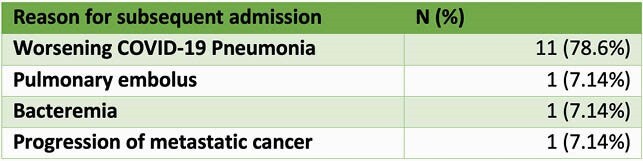

**Conclusion:**

Mortality rate in outpatients with severe COVID-19 treated with RDV was similar to that reported in inpatients. In this cohort of patients with severe COVID, a majority (84.1%) avoided hospitalization while still receiving appropriate treatment. Results suggest RDV can be safely delivered to outpatients with severe COVID-19.

**Disclosures:**

**All Authors**: No reported disclosures

